# Driving developments in UK oesophageal radiotherapy through the SCOPE trials

**DOI:** 10.1186/s13014-019-1225-0

**Published:** 2019-02-04

**Authors:** S. Gwynne, E. Higgins, A. Poon King, G. Radhakrishna, L. Wills, S. Mukherjee, Maria Hawkins, G. Jones, J. Staffurth, T. Crosby

**Affiliations:** 1 South West Wales Cancer Centre, Swansea, UK; 2 NIHR Cardiff RTTQA Group, Cardiff, UK; 3 Christie Hospital, Manchester, UK; 4 CRUK/MRC Oxford Institute for Radiation Oncology, Oxford, UK; 5 Cardiff University, Cardiff, UK; 6 Velindre Cancer Centre, Cardiff, UK

**Keywords:** Oesophagus, Radiotherapy, Quality assurance

## Abstract

**Background:**

The SCOPE trials (SCOPE 1, NeoSCOPE and SCOPE 2) have been the backbone of oesophageal RT trials in the UK. Many changes in oesophageal RT techniques have taken place in this time. The SCOPE trials have, in addition to adopting these new techniques, been influential in aiding centres with their implementation. We discuss the progress made through the SCOPE trials and include details of a questionnaire sent to participating centres. to establish the role that trial participation played in RT changes in their centre.

**Methods:**

Questionnaires were sent to 47 centres, 27 were returned.

**Results:**

100% of centres stated their departmental protocol for TVD was based on the relevant SCOPE trial protocol. 4DCT use has increased from 42 to 71%. Type B planning algorithms, mandated in the NeoSCOPE trial, were used in 79.9% pre NeoSCOPE and now in 83.3%.

12.5% of centres were using a stomach filling protocol pre NeoSCOPE, now risen to 50%. CBCT was mandated for IGRT in the NeoSCOPE trial. 66.7% used this routinely pre NeoSCOPE/SCOPE 2 which has risen to 87.5% in the survey.

**Conclusion:**

The results of the questionnaires show how participation in national oesophageal RT trials has led to the adoption of newer RT techniques in UK centres, leading to better patient care.

**Electronic supplementary material:**

The online version of this article (10.1186/s13014-019-1225-0) contains supplementary material, which is available to authorized users.

## Background

Chemoradiotherapy (CRT) has a role in the management of potentially curable oesophageal cancer in the definitive (dCRT) [1] and neoadjuvant (naCRT) settings [2]. The SCOPE trials (SCOPE 1 [3], NeoSCOPE [4] and SCOPE 2 [5]) have been the backbone of trials involving radiotherapy (RT) for oesophageal cancer in the UK over the last ten years. SCOPE 1 opened to recruitment in 2008, NeoSCOPE in 2013, and SCOPE 2 opened in early 2017. Many changes in oesophageal RT techniques have taken place in the intervening period. In the UK, most modern RT techniques implemented in departments across the country have been as the result of large scale randomised trials [6]. The SCOPE trials in oesophageal cancer have, in addition to adopting these new techniques, been influential in aiding centres with their implementation. Here we discuss the progress made through the SCOPE trials and include details of a questionnaire sent to participating centres to establish the role that trial participation played in RT changes in their centre.

## Methods

Questionnaires were provided to 47 centres that had participated in SCOPE 1/NeoSCOPE or expressed interest in SCOPE 2. Questionnaires were first distributed to participants at the SCOPE 2 launch meeting held in April 2016 and by email to those who were unable to attend the meeting. Reminder emails were sent between April 2016 and October 2017. By this time point 27 questionnaires were returned and the results are presented here. The results were analysed by question and no additional stratification was made for entry or timeline in working towards entry. The results refer to the use of the technique in routine clinical practice, rather than specifically for trial patients. The questionnaire is available in the Additional file 1 section.

### The SCOPE trials

SCOPE 1 [3] was a phase 2/3 multi-centre trial of CRT for patients with non-metastatic oesophageal cancer, incorporating the use of biological therapy. Patients were randomised to CRT alone or CRT with the addition of cetuximab. The CRT regimen consisted of two cycles of cisplatin and capecitabine followed by 2 further cycles of cisplatin and capecitabine with conformally planned RT to a dose of 50Gy in 25 fractions. A detailed trial protocol and QA programme [7] accompanied the trial.

NeoSCOPE [8] was a phase 2 multi-centre trial of naCRT prior to surgery for oesophageal cancer. Its aim was the re-introduction this regimen into the UK in the era of modern RT techniques and post-operative care, after its use was abandoned a decade previously, following concerns over morbidity and mortality [9], in addition to establishing the optimal neoadjuvant therapy in this disease. Patients received 2 cycles of neoadjuvant chemotherapy with capecitabine and oxaliplatin and were then randomised to one of two concurrent chemotherapy regimens: capecitabine and oxaliplatin or carboplatin and paclitaxel. This trial was also accompanied by a detailed RT protocol [4] and a quality assurance (QA) programme [10].

SCOPE 2 followed on from SCOPE 1 for the definitive non-surgical management of oesophageal cancer. The trial is looking at the role of dose intensification and adaptive therapy. Through a 2 × 2 design, one randomisation examines dose escalation from 50Gy in SCOPE 1 to 60Gy, to gross tumour, delivered as simultaneous integrated boost [11]. There is also a 2nd optional randomisation with systemic therapy adaptation based on PET response after one cycle.

The corresponding RT developments in each of the trials are detailed in Table 1. Centres were supported in implementing these developments through a comprehensive quality assurance programme [12].

**Table 1:** RT developments in each of the three SCOPE trials

RT development	Trial 1st introduced
Iv contrast for TVD	SCOPE 1
Consensus protocol for target volume delineation	SCOPE 1
3d conformal planning /single phase plan	SCOPE 1
EUS and PET for TVD	SCOPE 1
Use of prospective dose volume constraints	SCOPE 1
RTTQA programme	
Benchmark case for middle 1/3 only, retrospective review of selected on-trial cases	SCOPE 1
Benchmark case for both middle 1/3 and lower 1/3 case, real time review of 1st on-trial case and all cases up until toxicity assessment, timely retrospective review of all remaining on-trial cases, outlining atlas, outlining workshop	NeoSCOPE
Elective nodal irradiation	NeoSCOPE
Stomach filling protocol	NeoSCOPE
Type B algorithms	NeoSCOPE
CBCT	NeoSCOPE
4DCT	NeoSCOPE
IMRT/use of simultaneous integrated boost	SCOPE 2

## Results

There was a return rate of 57% in this study. There was good representation from departments across the UK, including the devolved nations. The results are shown in Table 2.

**Table 2:** Results of questionnaire

Development	Use prior to respective trials	Use currently	Comments
Consensus protocol for target volume delineation	Not specifically assessed in questionnaire	100%	Prior to SCOPE 1 trial there was wide variation in practice across the UK [14]
Use of prospective dose constraints	Not specifically assessed in questionnaire	96%	In 88% of centres these are based on the corresponding SCOPE trial
3d conformal planning	29%	100%	Now replaced by IMRT in SCOPE 2
Stomach filling protocol	12.5%	50%	
CBCT	66.7%	87.5%	
4D CT	42%	71%	
Use of type b algorithm	79.9%	83.3%	pre NeoSCOPE^a^
IMRT	75% [1]	79%	[1]12.5% only using for upper 1/3 pre SCOPE 2

^a^Only 14/36 (39%) benchmark cases in SCOPE 1 used a type be algorithm [12]

## Discussion

### Pre-treatment

#### Target volume delineation

Variation in target volume delineation (TVD) can be a major issue for clinical trials and without clear protocol clinicians will adopt their own interpretation of target volumes [13]. Prior to SCOPE 1 there was no consensus protocol around the UK for the use of dCRT for oesophageal cancer [14]. Access to a clear protocol has been shown to reduce variation in TVD [15]. Our survey showed that 24/24 (100%) centres stated that they had a departmental protocol for TVD and in all cases this was based on the relevant SCOPE trial protocol. One centre commented that TVD had become more uniform among clinicians through participation in the SCOPE trials.

EUS (endoscopic ultrasound_ was mandated, when the tumour was passable, through all the 3 SCOPE trials. PET (positron emission tomography) was considered optional for the SCOPE 1 [3] and NeoSCOPE trials [4] (Table 3) but was mandated in the SCOPE 2 trial. The role of PET in adapting the concurrent systemic therapy given concurrent with the RT is being investigated in SCOPE 2, with PET scans only being undertaken in approved centres to ensure consistency (http://www.ncri-pet.org.uk).

**Table 3:** 4DCT acquisition options in the NeoSCOPE trial

	4DCT method 1	4DCT method 2
Pre-delineation	From the 4DCT data sets, identify the extreme phases of motion (MaxIn and MaxEx). Also identify the phase that best represents the time-weighted average (Mid).	From the 4DCT data sets, identify the extreme phases of motion (MaxIn and MaxEx).
GTV	Contour as per the 3D protocol on each of the three phases as defined above, giving: GTV_MaxIn_, GTV_Mid_ and GTV_MaxEx._	Contour as per the 3D protocol on the 3D contrast enhanced CT scan and label it GTV_3D_. Also contour the GTV in the extreme phases of the 4D scan, giving GTV_MaxIn_ and GTV_MaxEx._ Combine these three to obtain a composite structure, label GTV_motion._ Review GTV_motion_ on all 4DCT phases and manually increase the contour for any areas not covered.
CTVA	Contour as per the 3D protocol on each of the three phases, giving: CTVA_MaxIn_, CTVA_Mid_ and CTVA_MaxEx._	Contour as per the 3D protocol on the 3D contrast enhanced CT scan using GTV_motion_ as the starting point. Label CTVA_3D._
CTVB	Contour as per the 3D protocol on each of the three phases, giving: CTVB_MaxIn_, CTVB_Mid_ and CTVB_MaxEx._	Create CTVB_3D_ from CTVA_3D_ as per the 3D protocol on the 3D contrast enhanced CT. Make two copies of CTVB_3D_, labelled CTVB_MaxIn_ and CTVB_MaxEx_ then proceed to manually increase these on their respective respiration phases for any areas not covered.
ITV	The ITV is defined as the composite CTVB volumes. Review the ITV on all 4DCT phases and manually increase the contour for any areas not covered.	The ITV is made by combining CTVB_MaxIn_ and CTVB_MaxEx_. Check that this volume covers any unusual motion patterns noted in the respiratory phases above.
PTV	Apply the margin to the ITV	Apply the PTV margin to the ITV
Planning	The mid phase CT is used for planning the dose distribution	The 3D CT is used for planning the dose distribution

Historically, there has been no evidence-based consensus regarding TVD in oesophageal cancer. Studies of dCRT and naCRT have used variable definitions of the extent of gross disease, elective lymph node irradiation (ELNI) and clinical target volume (CTV) and planning target volumes (PTV). Protocols for target volume delineation, planning technique and doses to OARs were developed for all the trials. CTV and PTV margins in SCOPE 1 were based on the distance from the gross tumour volume (GTV), using a combination of manual growth and computer-generated geometric margins, but NeoSCOPE and SCOPE 2 offered the opportunity to further refine the definition of ‘tissue at risk’ encompassed in the CTV. NeoSCOPE and SCOPE 2 attempted to define areas of elective nodal irradiation (ELNI) areas as part of the CTV [9]. For SCOPE 1 there was liaison with several leading centres to identify current best practice and agree minimum standards to be adopted UK wide. NeoSCOPE and SCOPE 2 TVD protocols were based on discussions at an Upper GI Radiotherapy Planning Workshop held in 2011 [9] and SCOPE2 launch meeting held in 2015, taking into account the EORTC-ROG Guidelines for neo-adjuvant radiation of adenocarcinomas of the GE junction and stomach [16] and UK Patterns of Failure work [17]. The target volumes in the SCOPE 2 trial are detailed in Table 4 .There were additional to opportunities to road test the protocols with members of the trial management group and to adapt the protocol based on the issues identified through the QA programme. This iterative approach to trial protocol development is now considered an essential component of ensuring consistency in TVD within trials [15].

**Table 4:** Target volumes for SCOPE 2

Structure name		Description	
GTVp		Includes: • Primary tumour (above or below GOJ) • Any involved nodes above the GOJ and ≤ 30 mm of the primary tumour volume • The circumference of the oesophagus at the level of disease	
GTVn		Includes any involved nodes below GOJ or > 30 mm from primary tumour (GTVp). It is not required to include the full circumference of the oesophagus.	
GTVpn		Union of GTVp and GTVn, and the circumference of the intervening oesophagus/proximal stomach, to include only GTVn above the diaphragm.	
CTVA		GTVpn extended along the axis of the oesophagus: • Sup-inf margin of 20 mm from the edges of GTVp or 10 mm from the highest/lowest involved lymph node (GTVn). • The whole circumference of the oesophageal wall should be included. • Inferiorly CTVA should not extend below the GOJ.	
CTVn		GTVn + 5 mm isotropically	
CTVB		CTVA + 10 mm circumferentially: • CTVB is edited to exclude lung, pericardium, large vessels, right and main bronchi, liver and the vertebrae, both above and below the diaphragm. DO NOT edit beyond CTVC. For T4 tumours, DO NOT edit the respective structure (e.g., if T4 right pleura, do not edit the CTVB extending into right lung). • *Below the GOJ* CTVB is grown manually to include the volume at risk to a total of 20 mm below GTVp and at least 10 mm below the lowest GTVn). This volume includes CTVn and the elective nodal regions at high risk of microscopic spread.	
CTVC		CTVA+ 5 mm circumferential margin.	
ITV (For distal tumours where 4DCT used)		Composite of CTV volumes (from reference 3D, max end-inhale and max end-exhale scans), grown to account for any additional motion seen from all other 4DCT phases.	
PTV_5000 (Standard and high dose arm)		Proximal Tumours (4DCT not permitted)	CTVB + 10 mm sup, 10 mm inf, 5 mm circumferentially.
		Distal tumours (4DCT not used)	CTVB + 10 mm sup, 15 mm inf, 5 mm circumferentially.
		Distal tumours (4DCT used)	ITV + 5 mm isotropically.
PTV_6000 (High dose arm only)	Proximal and distal tumours (where 4DCT not used)	GTVp + 5 mm isotropically	
	Distal tumours (4DCT used)	GTVp_Ref + 5 mm isotropically	

*GTV* gross tumour volume, *CTV* clinical target volume, *ITV* internal target volume, *PTV* planning target volume

#### 4DCT

Motion of the lower oesophagus/gastro-oesophageal junction primary and ELNI regions can be marked during respiration and subject to motion and deformation as a consequence of respiration, swallowing, peristalsis, gastric filling and emptying, vascular and cardiac pulsations [9]. The use of four-dimensional (4D) scanning has the potential to reduce the resulting risk of geographical miss, by accounting for this patient-specific variation over the course of a respiration cycle [18]. To our knowledge, NeoSCOPE was the first multicentre study to incorporate 4DCT and conferred the opportunity to introduce a standardised protocol for its acquisition and to prospectively evaluate its use within this feasibility study [9].

In order to facilitate centres to undertake 4DCT in the trial, the RT protocol gave 2 options for creation of an internal target volume (ITV) with 4DCT (see Table 3), reflecting the practice of two of the centres with the most experience in 4DCT for oesophageal RT at that point in time. Centres wishing to undertake 4DCT within the trial were encouraged to attend a workshop with break-out sessions for both physicists and clinicians, looking at issues surrounding scan acquisition and outlining respectively. A 4DCT pre-accrual test case was also made available for those who were not able to attend the workshop.

A single method of ITV creation was recommended for the SCOPE 2 trial (4DCT method 1). A Royal College of Radiologists (RCR) webinar (https://www.rcr.ac.uk/clinical-oncology/webinar-image-guided-radiotherapy-oesophageal-cancer) was developed to assist centres with implementing this technique. A 4DCT pre-accrual test case was also made available. Prior to NeoSCOPE only 10/24 (42%) of centres in our survey were using 4DCT for lower third oesophageal cases The number using 4DCT had increased to 17 (71%) by the time of the survey.

#### Stomach filling protocol

NeoSCOPE and SCOPE 2 introduced a stomach filling protocol for anatomical reproducibility [19]. Patients were asked to fast for 2 h and then drink 200mls of liquid 30 min prior to CT planning and treatment. Patients who had an NG tube inserted were advised to use their tube for this purpose. 12.5% of centres were using a stomach filling protocol prior to the NeoSCOPE and SCOPE 2 trials, which has now risen to 50%.

### Planning technique

#### 3D conformal radiotherapy

Prior to SCOPE 1 some centres were adopting a 2-phase approach to oesophageal RT with anterior posterior fields to 30Gy followed by a conformal volume to a total of 50Gy. SCOPE 1 mandated a single phase 3-Dimensional (3D) conformal RT and this became the standard of care in many centres for oesophageal cancer. The latter led to significant improvements in terms of cardiac dose-volume criteria [20, 21], in addition to considerable logistical advantages in workload [21]. 29% of centres stated they used a single 3D conformal plan prior to SCOPE 1. Current survey results showed that 100% of centres used a single conformal volume (unless using intensity modulated radiotherapy (IMRT), see below).

#### IMRT

 The first planning study looking at the dosimetric benefits of IMRT for oesophageal cancer was from the Royal Marsden in 2001 [22]. Due to concerns in the preoperative setting regarding low doses of RT to a large lung volume, NeoSCOPE did not allow the use of IMRT/volumetric modulated arc therapy (VMAT) [9], but it is mandated in the SCOPE 2 trial. IMRT improves dose conformity and reduces radiation exposure to normal tissues. Lin *et al* [23] compared long-term clinical outcomes in 2 large cohorts of oesophageal patients treated with 3D-CRT (*n* = 413) and IMRT (*n* = 263). Compared with IMRT, 3D-CRT patients had a significantly greater risk of dying (72.6% vs 52.9%, inverse probability of treatment weighting, log-rank test, *P* < .0001) and of locoregional recurrence (*p* = .0038). No difference was seen in cancer-specific mortality; however an increased cumulative incidence of cardiac death was seen in the 3D-CRT group (*p* = .049), suggesting IMRT should be considered for treatment of oesophageal cancer.

Planning studies from Oxford had shown that doses of RT could be escalated without exceeding doses to organs at risk when a simultaneous integrated boost to the GTV was delivered, using IMRT [11]. SCOPE 2 mandated IMRT for both the standard and dose escalated arms, where 3DCRT had been used in SCOPE 1 and NeoSCOPE. Although IMRT is available in most UK centres [24], it has not been routinely used for middle and lower third oesophageal cancers. 75% of centres stated they used IMRT for oesophageal cancer pre-SCOPE 2, but at least 3/24 (12.5%) centres stated they were only using for upper 1/3 oesophageal cancers, where the anatomy has made meeting dose constraints more difficult. 79% of centres in our survey now use IMRT for oesophageal tumours, including for middle and lower 1/3 tumours. Implementation of IMRT for new tumour sites has been assisted by the opportunity to receive feedback on a planning exercise and a credentialing programme for IMRT through the National Radiotherapy Trials Quality Assurance (RTTQA) group [25].

#### Planning algorithms

The choice of dose calculation algorithm for RT planning affects the accuracy of calculated.

dose distributions, particularly in areas of tissue inhomogeneity such as the lungs. In heterogeneous situations, the algorithms which provide approximate modelling for the variation of penumbra with density (type b) have been shown to model the dose more accurately than those which do not (type a) when compared to Monte Carlo as a gold standard of calculation accuracy. This is of importance in oesophageal RT as it is often the case that the gross disease lies adjacent to the lung tissue [26]. While both of the algorithms were in use at the time of the SCOPE 1 trial, type b algorithms were limted by computational power and the need to clinically implement this change. NeoSCOPE mandated the use of type b algorithm for determining lung dose after work from SCOPE 1 showed it superiority to type a algorithm [26]. Type b planning algorithms mandated in the NeoSCOPE trial, were used were used in 39% of SCOPE 1 pre-accrual planning case [12], rose to 79.9% by the time of the NeoSCOPE trial and now used in 83.3% of centres. SCOPE 2 will also mandate type b algorithms.

### Dose volume constraints

Prior to SCOPE 1 only 42% outlined the heart as an organ at risk (OAR) [14]. The heart was mandated as an OAR and a dose constraint detailed in the RT protocol. In order to assist with the delineation of the heart as an OAR an atlas was included within the radiotherapy guidance document, which was adopted for use in some UK breast trials also [12]. In our survey 96% of centres had pre-defined dose volume constraints for oesophageal RT and in 88% these were based on the corresponding SCOPE trial. The dose constraints for SCOPE 2 are shown in Table 5.

**Table 5:** Dose constraints in the SCOPE 2 trial

Structure name	Constraint	Optimal	Mandatory
PTV_6000 (High dose arm only)	V95% (57Gy)	> 95%	≥ 90%
	Dmedian	100% (60Gy)	The median should be between 98 and 102% of the prescription dose (i.e., 60Gy).
PTV_5000	V95% (47.5Gy)	> 95%	≥ 90%
	Dmedian	100% (50Gy, standard dose arm only)	The median should be between 98 and 102% of the prescription dose (i.e., 50Gy, standard dose arm only).
External	D1.8 cc		< 107% of highest prescribed dose
SpinalCord_PRV	D0.1 cc	< 40Gy	< 42 Gy
Heart	Dmean	< 25Gy	*<30Gy*
	V30Gy	< 45%	*-*
Lungs	Dmean	< 17Gy	<19Gy
(Combined lungs)	V20Gy	< 20	≤25
Stomach_excl_PTV_5000 (Stomach excluding PTV_5000)	V50Gy	< 16 cc	< 25 cc
Liver	Dmean	≤28Gy	≤30Gy
	V30Gy	V30Gy < 30%	-
Kidney_L and Kidney_R (Individual kidneys)	V20Gy	< 25%	≤30%

### Treatment verification

#### IGRT

TVD with 4DCT, by taking into account internal motion, leads to individualised margins, but needs to be accompanied by rigorous quality control of treatment delivery. Two-dimensional megavoltage portal imaging is insufficient in the 4D era, where a form of volumetric imaging, such as cone-beam computed tomography (CBCT) is required [9, 27]. CBCT images are obtained with the patient in the treatment position and then matched to the planning scan via automated software. Studies have shown the reliability of this technique in the treatment of oesophageal and gastrooesophageal cancers [27, 28]. CBCT was mandated for IGRT in the NeoSCOPE trial. 66.7% used this routinely pre NeoSCOPE/SCOPE 2 which has risen to 87.5% in the survey.

#### RTTQA

SCOPE 1 introduced a Radiotherapy Quality Assurance (RT QA) programme to ensure the quality of the RT delivered in the trial [12], as it is now well documented that protocol deviations can effect outcome [29]. This took place under the auspices of the UK National Radiotherapy Trials Quality Assurance (RTTQA) Group [30]. A pre-accrual outlining and planning exercise of a middle third tumour was undertaken by all participating centres [7]and a detailed RT protocol provided, which included an atlas of worked examples and heart outlining [12]. Centres were required to pass this exercise before entry into the trial. The results of the outlining part of this exercise have been published elsewhere [7, 12]. On-trial centres were required to send both a completed PAF and the anonymised full DICOM data, consisting of the planning CT images, structure set, plan and calculated dose for every patient. A retrospective analysis of on-trial patients was planned on the 1st case from each centre and after that a 10% random sample [12].

For NeoSCOPE, in part due to the concerns raised about possible toxicity, there was a more rigorous QA process [4]. An outlining workshop was held, an outlining atlas developed and an outlining workshop held. Prior to trial participation centres were again required to satisfactorily complete a pre-accrual benchmark outlining and planning case. Investigators were asked to outline both a middle and lower 1/3 oesophageal case and to plan a pre-outlined lower 1/3 case, both according to the protocol. Individual case review was carried out prospectively for the 1st case from each centre and for the first 20 cases, repeating the process if there was an issue, until there was a satisfactory submission. Feedback to centres was within 3 working days. For all remaining cases we used ‘timely retrospective review’, where outlines and plans were reviewed within 2 weeks of the start of radiotherapy, allowing the identification and correction of major issues before the treatment was complete. As a result all 85 cases in the NeoSCOPE trial had some form of contemporaneous QA. The results of this process have been presented elsewhere [31, 32]. This was facilitated by the development of a compiled viewing package enabling reviewers from multiple centres to undertake the reviews [10].

For SCOPE 2, as per NeoSCOPE trial, a rigorous RTQA programme was established. There was however a move within the UK RTTQA community to reduce the burden of QA on centres by streamlining the QA process for trials as far as possible, so centres that had previously passed the pre-accrual outlining QA for NeoSCOPE were exempt from repeating the pre-accrual outlining exercise. Due to a change in planning technique we were unable to do the same for the pre-accrual planning exercise. A detailed RT protocol was again produced and subjected to an iterative development process after peer review. As with NeoSCOPE, it is expected that pre-accrual QA will be accompanied by an ‘on trial’ component with a mixture of both real time (feedback within 3 working days) and timely retrospective review (feedback within first 10 fractions of RT). Due to the number of cases within the trial real time QA will be reserved for the 1st case from each centre (+/− 1st high dose arm) of all cases treated within the trial.

The RTTQA process for the trials has led to the establishment of a community of practice with 2 way dialogues with centres providing a supportive, mentoring culture which has been shown to support learning [33]. An educational sub study is being undertaken within the SCOPE 2 trial to evaluate this further. 33% of centres stated that they had not had any form of peer review of their outlining before participation in the SCOPE trials with 88% reporting that they found this process helpful. There were comments from those woking in smaller grateful for the peer review. The RCR plans to address the need to peer review of outlining of non-trial patients with recently published guidance (https://www.rcr.ac.uk/clinical-oncology/service-delivery/radiotherapy-target-definition-and-peer-review), which has drawn heavily in some aspects on the experience of trials QA in the SCOPE trials.

## Conclusion

Accepting the limitations of the study (57% return rate, single time point and risk of recall bias), the results of the questionnaires support how participation in national oesophageal RT trials has led to the adoption of newer RT techniques in UK centres, leading to better patient care. These developments have been supported by a comprehensive quality assurance programme. Most importantly, it has shown that there is an enthusiastic upper gastrointestinal clinical oncology community that can successfully complete trials and deliver high-quality RT.

## Additional file

Additional file 1: Supplementary material Questionnaire sent to centres. (DOC 27 kb)

## Abbreviations

3D: 3-Dimensional
4D: Four-dimensional
CRT: Chemoradiotherapy
CTV: Clinical target volume
dCRT: Definitive
ELNI: Elective lymph node irradiation
EUS: Endoscopic ultrasound
GTV: Gross tumour volume
IMRT: Intensity modulated radiotherapy
ITV: Internal target volume
naCRT: Neoadjuvant (naCRT)
OAR: Organ at risk
PET: PET (positron emission tomography
PTV: Planning target volume
QA: Quality assurance (QA)
RCR: Royal College of Radiologists
RT: Radiotherapy (RT)
RTTQA: Radiotherapy Trials Quality Assurance
TVD: Target volume delineation
VMAT: Volumetric modulated arc therapy

## References

[1] Rackley T, Leong T, Foo M (2014). Definitive Chemoradiotherapy for Oesophageal Cancer, a promising start on an exciting journey. Clin Oncol.

[2] Gwynne S, Wijnhoven B, Hulshof M (2014). Role of Chemoradiotherapy in Oesophageal Cancer - adjuvant and neoadjuvant therapy. Clin Oncol.

[3] Crosby T, Hurt C, Falk S (2013). Chemoradiotherapy with or without cetuximab in patients with oesophageal cancer (SCOPE1): a multicentre, phase 2/3 randomised trial. Lancet.

[4] Mukherjee S, Hurt C, Gwynne S (2015). NEOSCOPE: a randomised Phase II study of induction chemotherapy followed by either oxaliplatin/capecitabine or paclitaxel/carboplatin. based chemoradiation as pre-operative regimen for resectable oesophageal adenocarcinoma. BMC Cancer.

[5] SCOPE 2 Trials. 2017. https://clinicaltrials.gov/ct2/show/NCT02741856. Accessed Jan 2019.

[6] K V, Tsang YCL, Coles CE, Yarnold JR (2012). Does participation in clinical trials influence the implementation of new techniaues? A look at changing techniques in breast radiotherapy in the UK. Clin Oncol.

[7] Gwynne S, Spezi E, Wills L (2012). Inter-observer variation in outlining of pre-trial test case in the SCOPE1 trial: a United Kingdom definitive Chemoradiotherapy trial for esophageal Cancer. Int J Radiat Oncol Biol Phys.

[8] Mukherjee S, Hurt C, Gwynne S (2017). NEOSCOPE: a randomised phase II study of induction chemotherapy followed by oxaliplatin/capecitabine or carboplatin/paclitaxel based pre-operative chemoradiation for resectable oesophageal adenocarcinoma. Eur J Cancer.

[9] Gwynne S, Falk S, Gollins S (2013). Oesophageal Chemoradiotherapy in the UK - current practice and future directions. Clin Oncol.

[10] Gwynne S, Jones G, Maggs R (2016). Prospective review of radiotherapy trials through implementation of standardized multicentre workflow and IT infrastructure. Br J Radiol.

[11] Warren S, Partridge M, Carrington R (2014). Radiobiological determination of dose escalation and normal tissue toxicity in definitive chemoradiation therapy for esophageal cancer. Int J Radiat Oncol Biol Phys.

[12] Wills L, Maggs R, Lewis G (2017). Quality assurance of the SCOPE 1 trial in oesophageal radiotherapy. Radiat Oncol.

[13] Hong T, Toméb W, Hararib P (2012). Heterogeneity in head and neck IMRT target design and clinical practice. Radiother Oncol.

[14] Button MR, Staffurth J, Crosby TDL (2007). National variations in the treatment of oesophageal cancer with chemoradiotherapy. Clin Oncol.

[15] Gwynne S, Spezi E, Sebag-Montefiore D (2013). Improving radiotherapy quality assurance in clinical trials: assessment of target volume delineation of the pre-accrual benchmark case. Br J Radiol.

[16] Matzinger O, Gerber E, Bernstein Z (2009). EORTC-ROG expert opinion: Radiotherapy volume and treatment guidelines for neoadjuvant radiation of adenocarcinomas of the gastroesophageal junction and the stomach. Radiother Oncol.

[17] Button MR, Morgan CA, Croydon ES (2009). Study to determine adequate margins in radiotherapy planning for esophageal carcinoma by detailing patterns of recurrence after definitive chemoradiotherapy. Int J of Radiat Oncol Biol Phys.

[18] Zhao K, Liao Z, Bucci MK (2007). Evaluation of respiratory-induced target motion for esophageal tumors at the gastroesophageal junction. Radiother Oncol.

[19] Matthews E, Bowden C, Nicholas O, et al. Radiation Therapy for Cancer of the Oesophagus and Pancreas, SMGroup, 2016. http://www.smgebooks.com/Radiation-Oncology/chapters/RADO-16-03.pdf.

[20] Mukherjee S, Aston D, Minett M (2003). The significance of cardiac doses received during Chemoradiation of Oesophageal and gastro–oesophageal junctional cancers. Clin Oncol.

[21] Wills L, Lewis G, Passant H (2005). A Single-phase Versus Two-phase Conformal Planning Study for Oesophageal Radiotherapy. Clin Oncol.

[22] Nutting CM, Bedford JL, Cosgrove VP (2001). A comparison of conformal and intensity modulated techniques for oesophageal radiotherapy. Radiother Oncol.

[23] Lin SH, Wang L, Myles B (2012). Propensity score-based comparison of long-term outcomes with 3-dimensional conformal Radiotherapy vs intensity-modulated Radiotherapy for esophageal Cancer. Int J Radiat Oncol Biol Phys.

[24] Staffurth J (2010). A review of the clinical evidence for intensity-modulated Radiotherapy. Clin Oncol.

[25] Eaton D, Tyler J, Backshall A (2017). An external dosimetry audit programme to credential static and rotational IMRT delivery for clinical trials quality assurance. Phys Med.

[26] Wills L, Millin A, Paterson J (2009). The effect of planning algorithms in oesophageal radiotherapy in the context of the SCOPE 1 trial. Radiother Oncol.

[27] Hawkins MA, Aitken A, Hansen VN (2011). Set up errros for oesophageal cancers - is electronic portal imaging or conebeam more accurate?. Radiother Oncol.

[28] Hawkins MA, Brooks C, Hansen VN (2010). Cone beam computed tomography-derived adaptive radiotherapy for radical treatment of esophageal cancer. Int J Radiat Oncol Biol Phys.

[29] Weber DC, Tomsej M, Melidis C (2012). QA makes a clinical trial stronger: evidence-based medicine in radiation therapy. Radiother Oncol.

[30] Miles E (2010). A co-ordinated approach to national QA for multi Centre clinical trials: the UK RT trials QA group. Radiother Oncol.

[31] Rackley T, Caley A, Spezi E, et al. Oesophageal delineation - lessons learnt from pre-accrual benchmark cases in the UK NeoSCOPE oesophageal trial. Int J Radiat Oncol Biol Phys. 2014;90:S10.

[32] Evans E, Jones G, Radhakrishna G (2016). On-trial radiotherapy quality assurance in NeoSCOPE: a randomised phase II trial of chemoradiotherapy in oesophageal cancer. J Clin Oncol.

[33] Lave J, Wenger E. Communities of practice: learning, meaning and identity. Cambridge University Press; 1998.

