# Supplementary material for: Driving developments in UK oesophageal radiotherapy through the SCOPE trials
Source: Radiat Oncol. 2019 Feb 4;14:26. doi: 10.1186/s13014-019-1225-0 (PMC6360789; doi:10.1186/s13014-019-1225-0)
Supplement: Supplementary file 1 — Supplementary material Questionnaire sent to centres. (DOC 27 kb) [file 13014_2019_1225_MOESM1_ESM.doc]

Questionnaire to SCOPE trial participants

**Target volume delineation**

1a. Do you have a departmental protocol for outlining oesophageal cancer

Yes

No

1b. Are these based on the corresponding SCOPE trial e.g. SCOPE1 /2 for dCRT, NeoSCOPE for NA CRT?

Yes

No

2a. Do you use 4DCT for oesophageal cancer?

Yes

No

2b. Did you use 4DCT prior to the NeoSCOPE/SCOPE 2 trial

Yes

No

3a. Have you ever had peer review of your oesophageal volumes prior to your participation in the RTQA programme for the SCOPE trials?

No

Yes

3b. Have you found this review process helpful?

Yes

No

3c. Would you be willing to support extension of RTQA for outlining into training and service delivery?

Yes

No

3d.. If you have any specific comments to make about any aspect of RTQA please include them here

**Radiotherapy planning and treatment delivery**

4a. Do you have a departmental dose volume constraints for oesophageal cancer

Yes

No

4b. Are these based on the corresponding SCOPE trial e.g. SCOPE1 /2 for dCRT, NeoSCOPE for NA CRT?

Yes

No

5a. Do you use IMRT for oesophageal cancer?

Yes

No

5b. Did you use IMRT for this site prior to SCOPE 2?

Yes

No

6a. Have you had peer review of your oesophageal radiotherapy planning prior to the SCOPE trials?

Yes

No

6b. Have you found this review process helpful?

6c. Would you be willing to support extension of RTQA for planning into training and service delivery?

Yes

No

6d.. If you have any specific comments to make about any aspect of RTQA please include them here

7a. Do you use a gastric filling protocol for oesophageal cancer radiotherapy?

Yes

No

7b. Were you using this prior to the NeoSCOPE/SCOPE 2 trials?

Yes

No

8a. Are you using cone beam CT for verification?

Yes

No

8b. Were you using this prior to the NeoSCOPE/SCOPE 2 trials

Yes

No

9a. Are you using type B algorithms for planning?

Yes

No

9b. Were you using this prior to NeoSCOPE/SCOPE 2 trials

Yes

No

10. Were you using single phase conformal technique for oesophageal planning prior to SCOPE 1?

Yes

No

NA ( did not participate in SCOPE 1)

11.Are there any other areas of practice that have changed as a result of your centre’s participation in SCOPE trials?

Please detail them here
